# Referral Decision Making of General Practitioners: A Signal Detection Study

**DOI:** 10.1177/0272989X18813357

**Published:** 2018-12-27

**Authors:** Olga Kostopoulou, Martine Nurek, Simona Cantarella, Grace Okoli, Francesca Fiorentino, Brendan C. Delaney

**Affiliations:** Department of Surgery and Cancer, Faculty of Medicine, Imperial College London, London, UK; Department of Surgery and Cancer, Faculty of Medicine, Imperial College London, London, UK; Department of Surgery and Cancer, Faculty of Medicine, Imperial College London, London, UK; School of Population Health and Environmental Sciences, Faculty of Life Sciences and Medicine, King’s College London, London, UK; Department of Surgery and Cancer, Faculty of Medicine, Imperial College London, London, UK; Department of Surgery and Cancer, Faculty of Medicine, Imperial College London, London, UK

**Keywords:** cancer referral, conversion rate, detection rate, primary care, signal detection theory

## Abstract

**Background.** Signal detection theory (SDT) describes how respondents categorize ambiguous stimuli over repeated trials. It measures separately “discrimination” (ability to recognize a signal amid noise) and “criterion” (inclination to respond “signal” v. “noise”). This is important because respondents may produce the same accuracy rate for different reasons. We employed SDT to measure the referral decision making of general practitioners (GPs) in cases of possible lung cancer. **Methods.** We constructed 44 vignettes of patients for whom lung cancer could be considered and estimated their 1-year risk. Under UK risk-based guidelines, half of the vignettes required urgent referral. We recruited 216 GPs from practices across England. Practices differed in the positive predictive value (PPV) of their urgent referrals (chance of referrals identifying cancer) and the sensitivity (chance of cancer patients being picked up via urgent referral from their practice). Participants saw the vignettes online and indicated whether they would refer each patient urgently or not. We calculated each GP’s discrimination (*d* ′) and criterion (*c*) and regressed these on practice PPV and sensitivity, as well as on GP experience and gender. **Results.** Criterion was associated with practice PPV: as PPV increased, GPs’*c* also increased, indicating *lower* inclination to refer (*b* = 0.06 [0.02–0.09]; *P* = 0.001). Female GPs were more inclined to refer than male GPs (*b* = −0.20 [−0.40 to −0.001]; *P* = 0.049). Average discrimination was modest (*d*′ = 0.77), highly variable (range, −0.28 to 1.91), and not associated with practice referral performance. **Conclusions.** High referral PPV at the organizational level indicates GPs’ inclination to avoid false positives, not better discrimination. Rather than bluntly mandating increases in practice PPV via more referrals, it is necessary to increase discrimination by improving the evidence base for cancer referral decisions.

Earlier diagnosis of cancer has been part of the strategy for improving cancer outcomes in England since 2000.^1^ This includes the introduction of an urgent referral pathway: patients referred urgently for suspected cancer by a general practitioner (GP) are seen in specialist care within the national target of 2 weeks (the “2-week-wait” referral pathway). However, wide variation between general practices (“family practice clinics” in the United States) has been reported in relation to the quality of urgent cancer referrals across England. Meechan and colleagues^2^ used the national Cancer Waiting Times database and measured the between-practice variation in the proportion of urgently referred patients who were found to have cancer. This is the positive predictive value (PPV) of a practice’s referrals (Meechan and colleagues called this the “conversion rate”). They also measured the variation in the proportion of cancers diagnosed in a year that had been urgently referred by the practice. This is the sensitivity of a practice’s referrals (Meechan and colleagues called this the “detection rate”). The researchers suggested that a combination of high PPV and high sensitivity indicates “good clinical practice.” However, there are no data at the individual GP level on which to base this suggestion.

Meechan and colleagues also found an inverse relationship between practice PPV and referral ratio (i.e., the number of urgent referrals relative to the number of registered patients). Specifically, practices with low referral PPVs tended to have high referral ratios—that is, they referred many patients. By referring many patients, a practice would detect more cancers but also have more false positives—patients referred urgently who turn out not to have cancer. Thus, the low referral PPV of these practices may stem from a high rate of false positives. Furthermore, the researchers found a positive association between sensitivity and referral ratio: by referring more patients, practices detect more and miss fewer cancers, thereby boosting sensitivity. Both associations suggest that differences in the quality of practice referrals may be due to the GPs’ tendency to refer more or fewer patients. Nevertheless, the relationship between practice referral performance and individual GP decision making has never been studied; neither has the GPs’ ability to discriminate between patients who should and should not be referred ever been measured. We employed signal detection theory (SDT) to answer these questions.

SDT is a statistically based decision theory that describes how a decision maker categorizes ambiguous stimuli over repeated trials.^3^ To apply SDT to cancer detection, we need to assume that a GP is judging a patient on a decision variable (need for referral). Repeated presentations generate a distribution of likelihood values for referral in “signal” and “noise” cases. We can call these cases positive (patients needing referral) and negative (patients who should not be referred). The commonest model of SDT, the Gaussian equal variance model, assumes that these probability distributions are normal and with equal variance (Figure 1). On average, presentation of positive cases produces higher values on the decision variable than negative cases. However, the probability distributions overlap because some values on the decision variable can result from either type of case. On each trial, the GP decides whether referral is warranted by using a response “criterion.” If perceived need for referral falls above the criterion, the GP decides to refer; otherwise, he or she does not. Moving the criterion along the decision variable axis in Figure 1 can change the proportion of the 4 decision outcomes: hits, misses, correct rejections, and false alarms. Importantly, raising the criterion (moving right on the X-axis) will reduce both hits and false alarms, while lowering the criterion will increase them both.

**Figure 1:**
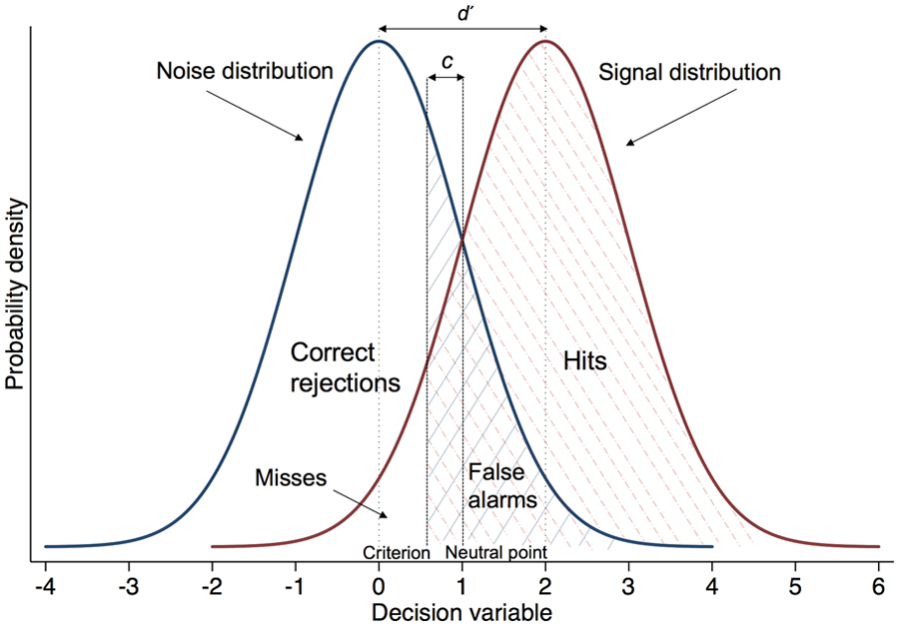
Hypothetical probability distributions of a decision variable, in units of standard deviation, across signal and noise trials. *M*_noise_ = 0, *M*_signal_ = 2, and *d* ′ = 2.

According to SDT, variation in accuracy can stem from 2 factors, “discrimination” and “response bias.” Discrimination or “sensitivity” is the respondent’s ability to discriminate signal from noise. It is quantified by the index *d* ′ (*d* prime), which is the standardized distance between the means of the signal and noise distributions. To calculate *d* ′, we need to know only a respondent’s hit rate (*H*) and false alarm rate (*FA*). The formula for *d* ′ is as follows:

$$d^{\prime }=z(H)-z(FA),$$

where *z* is the inverse of the cumulative normal distribution function.

Discrimination depends both on stimulus properties and the decision maker’s ability. In Figure 1, *d* ′ is 2. The larger the separation between the 2 distributions, the larger the *d* ′. A *d* ′ of 0 means no discrimination, while a *d* ′ of 4 is usually considered ceiling performance.

Response bias is the respondent’s inclination to make positive (yes/refer) v. negative (no/do not refer) decisions. It can be quantified by the index *c*, which is the distance between the respondent’s criterion location and the “neutral point” on the decision variable axis, where misses and false alarms are weighed equally, assuming equal base rates of events (Figure 1).^i^ A *c* value of 0 thus indicates no bias (the criterion is located on the neutral point). Negative values of *c* indicate a liberal approach and bias toward “yes” responses. Positive values of *c* indicate a conservative approach and bias toward “no” responses; the decision maker requires more evidence before acknowledging a “signal.” The formula for *c* is as follows:

$$c=-\frac{1}{2}\left[z(H)+z(FA)\right],$$

where *z* is the inverse of the cumulative normal distribution function. Thus, *c* is a measure derived from observed responses, not a value that the respondent decides to set on the decision variable. The optimal criterion (i.e., the criterion that maximizes the utility of the decision) depends on the base rate of the signal and how the decision maker evaluates the expected outcomes (costs and benefits).^4^ While discrimination is fixed, decision makers can alter their criterion by changing their willingness to respond “yes” v. “no.”

It is important to measure discrimination and response bias separately and not confound them since different people can produce the same accuracy rate for different reasons. Furthermore, discrimination and response bias can be targeted using different measures: instructions, mandates, and incentives can target response bias, while discrimination can improve only by providing decision makers with better evidence that separates signal from noise, reducing the overlap between the 2 distributions.

SDT has been used extensively to study performance on perceptual and more complex cognitive tasks,^5,6^ including medical diagnosis^4^ and clinical decision making.^7,8^ It has been used to study judgment biases of professionals,^9^ as well as biases at the level of systems/policies,^10^ to measure changes in risk perception following training^11^ and to derive optimal decision thresholds for controversial practices such as the use of physical constraints on psychiatric patients.^12^

## Aims and Hypotheses

We employed a signal detection approach to measure GPs’ discrimination and response bias when patients present with symptoms that could suggest lung cancer. By recruiting GPs from practices representing a range of referral performance, we aimed to explore how practice variation, as measured by Meechan and colleagues,^2^ is related to individual GPs’ decision making. We also aimed to explore the relationship between GP characteristics—namely, gender and years in general practice—and their referral decision making. A signal detection study of transfer decisions for trauma patients found that male emergency physicians had higher decision thresholds than their female counterparts.^8^

We had 2 hypotheses about GP criterion, based on the relationships between practice referral PPV and sensitivity with practice referral ratio reported by Meechan and colleagues^2^:

Hypothesis 1: There is a positive relationship between practice PPV and GP criterion, so that higher practice PPV is associated with a higher GP criterion (more conservative approach to referral).
Hypothesis 2: There is an inverse relationship between practice sensitivity and GP criterion, so that higher practice sensitivity is associated with a lower GP criterion (less conservative approach to referral).

Referral ratios are related to response bias (a high referral ratio suggests a liberal approach to referral) but not to discrimination. Therefore, we did not have strong hypotheses regarding associations between practice referral performance and GPs’ discrimination.

## Methods

### Sample Size and Recruitment

We used publicly available data from Public Health England to assign general practices to groups based on their 5-year (2011–2016) cancer referral PPV and sensitivity. Following the approach by Meechan and colleagues,^2^ we dichotomized and crossed practice PPV and sensitivity to have 4 practice types: low PPV and low sensitivity (LL), high PPV and high sensitivity (HH), low PPV and high sensitivity (LH), and high PPV and low sensitivity (HL). To dichotomize PPV, we calculated the median PPV per decile of sensitivity and took the mean of the resulting 10 medians (mean = 8.80%). Likewise, to dichotomize sensitivity, we calculated the median sensitivity per decile of PPV and took the mean of the resulting 10 medians (mean = 46.63%).

We used G*Power 3.1 to calculate sample size.^13^ To detect a small effect size (Cohen’s *f* ^2^ of 0.05) in multiple linear regressions with 2 predictors (regressing discrimination and response bias on practice PPV and sensitivity) with 80% power and α = 0.05, we would need a minimum sample size of 196 GPs. We aimed to recruit an equal number of GPs from each practice type (LL, LH, HL, and HH).

We e-mailed 566 GPs who had participated in previous studies by our research group. We gave them a brief explanation of the study and encouraged them to forward the e-mail to their colleagues. Interested GPs could complete an online expression-of-interest form, asking for their unique practice code. This code was used to allocate each potential participant to a practice type. GPs were invited to participate until the quota for each practice type was met.

### Materials

We aimed to present GPs with a relatively representative sample of patients for whom lung cancer could be considered. We did not aim to present them with cancer v. noncancer patients, as the behavior of interest was referral, not diagnosis. To this purpose, we prepared 44 clinical vignettes: brief descriptions of patients presenting to the GP with either a single symptom persisting over 2 consecutive consultations or a pair of symptoms in the same consultation. The symptoms used were cough, fatigue, appetite loss, weight loss, and raised platelets. We did not use hemoptysis, a “red flag” for lung cancer, as we expected that it would lead to immediate referral. The symptom likelihood ratios (LRs) came from a case-control study based in primary care, which included 247 lung cancer patients and 1235 controls, matched for age, sex, and general practice.^14^ The study captured both coded and free-text symptoms via detailed case note review and calculated PPVs for single symptoms and symptom pairs. It was a major source of evidence for the 2015 National Institute for Health and Care Excellence (NICE) guidelines for suspected lung cancer.^15^

We used a population risk prediction model^16^ to calculate the prior odds of each patient getting lung cancer based on age and smoking. We then multiplied the prior odds by the LRs of each patient’s symptoms to get the posterior odds and converted them to the posterior 1-year lung cancer risk.

A chest X-ray result could be requested in all the vignettes. We limited to 2 the number of vignettes with an abnormal result, since we expected that it would lead to immediate referral. NICE’s recommended threshold for referral is 3%.^15^ To account for the additional diagnostic information of the chest X-ray, we applied an LR of 0.45 for the normal result^17^ and estimated that a minimum lung cancer risk of 6.43% would be needed to justify referral (see online Appendix 1 for the calculations). Half of our vignettes (22) had posterior risk <6.43% (negative cases); the other half had posterior risk >6.43% and warranted urgent referral (positive cases). Posterior risk ranged from 0.03% to 14.23% across vignettes, with median 3.09% for negative and 7.78% for positive cases.

We used the Qualtrics platform (Qualtrics, Provo, Utah) to present the vignettes online. To minimize response fatigue, we divided the vignettes in 2 parts (part A and part B), to be completed on different days (see Procedure). The order in which the 2 parts were seen was randomized per GP and counterbalanced across GPs, so that each part was seen first and second an equal number of times.

### Piloting

We piloted different versions of the vignettes in 3 stages. Initially, 4 GPs completed 10 vignettes on paper in the presence of a researcher. They requested a chest X-ray every time, which led to our decision to include the chest X-ray option, as detailed earlier. At a second stage, 8 GP trainees completed 20 vignettes online and were invited to make comments after each vignette. Symptom duration varied between 2 and 6 weeks, which led to different perceptions of symptom severity; that is, symptoms of shorter duration were sometimes not considered serious, while those of longer duration were, and this differed between respondents, as evidenced by their written comments. Thus, we decided to hold symptom duration constant at 1 month. At the final stage of piloting, 2 GPs responded to the 44 vignettes in their current form online. They frequently used a free-text box labeled “working diagnosis” at the end of each vignette to write comments. To separate working diagnoses from comments for data analysis, we added an optional comments box at the end of each vignette. The GPs complained about response fatigue and loss of concentration; thus, we enabled vignette completion on 2 separate occasions, as detailed above.

### Procedure

**Figure 2:**
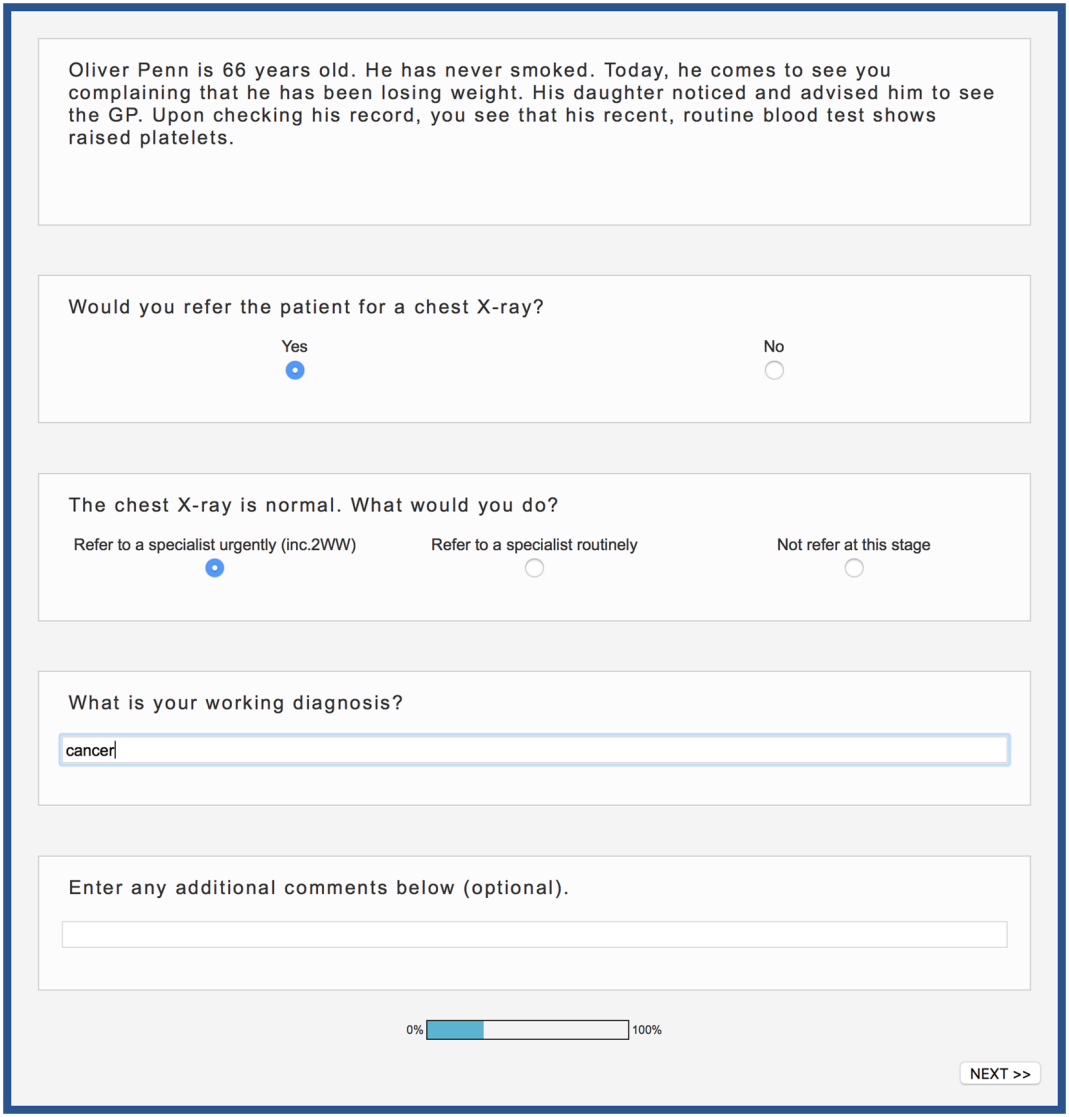
Screenshot of a vignette and the questions asked. All questions were compulsory, except for the last one. To avoid order effects, general practitioners (GPs) were randomly assigned to view the response categories in 1 of 2 orders: order 1 (depicted here) presented the response categories in the order of “yes,”“no” (chest X-ray question) and “refer to specialist urgently,”“refer to specialist routinely,”“not refer at this stage” (referral question). Order 2 presented the response categories in the opposite order for both questions. Order was held constant for a given GP across parts A and B.

Eligible participants who had filled in the expression-of-interest form were e-mailed a link to the study website. There, they read an information sheet, which explained that the study aimed to understand variation in GPs’ referral decisions but did not mention cancer. After providing informed consent online, participants completed the first part (either A or B), which comprised 22 vignettes (11 positive and 11 negative cases) presented in a random order. Figure 2 presents a screenshot of a vignette and the questions that GPs were asked. Each question appeared sequentially below the one just answered. Where the chest X-ray result was abnormal, this was made salient in red font. Twenty-four hours after completing the first part, GPs were e-mailed a link to the other part with the remaining 22 vignettes. Each part was estimated to take approximately 20 minutes to complete. Upon completion, participants were reimbursed £120, received a certificate for their portfolio of professional development, and were provided individualized feedback on their responses to each vignette.

### Analyses

For each GP, we counted the number of hits (positive cases urgently referred), misses (positive cases not urgently referred—either not referred at all or referred routinely), false alarms (negative cases urgently referred), and correct rejections (negative cases not urgently referred). We used each GP’s hit and false alarm rates to calculate their *d* ′ and *c*. When the number of either hits or false alarms is 0, *d* ′ and *c* cannot be calculated, and a correction is needed. As recommended in the literature, we added 0.5 to all data cells for all participants and calculated *d* ′ and *c* using the corrected values.^3,18^

We performed univariate linear regressions to test the effect of practice PPV, practice sensitivity, GP experience, and GP gender on discrimination (*d* ′) and criterion (*c*). We report the multivariable models with the significant predictors only.

An alternative way of estimating SDT indices is to use a generalized linear modeling (GLM) approach to regress the response (referral v. no referral) on the true class of the stimulus (positive v. negative case).^19^ Covariates can be entered to test their simultaneous influence on the SDT indices (coefficient and intercept of the regression). We checked the consistency of our estimates and conclusions derived from the traditional SDT approach with those derived from the GLM approach (online Appendix 2).

To test the robustness of our results to the risk threshold used to define the positive and negative cases (6.43%), we repeated all the analyses using 1) the unmodified NICE 3% risk threshold and 2) 2 new risk thresholds, a lower and an upper, which we estimated by using the lower and upper 95% confidence intervals for the original symptom LRs; we calculated these confidence intervals following a method recommended by Katz et al.^20^

Finally, to explore potential causes of misses and false alarms, we identified the 5 most frequent misses and the 5 most frequent false alarms. We analyzed thematically the free-text responses (working diagnoses and comments) associated with them. In parallel, we performed multilevel logistic regressions with random intercept for GPs on misses and false alarms separately, using risk factors (age, smoking status, pack years) and presenting symptoms as predictors. We used single symptoms rather than pairs in the regression models, since certain symptom pairs appeared only once across vignettes. STATA 13.1 (StataCorp LP, College Station, TX) was used for all statistical analyses.

## Results

The expression-of-interest form was completed by 467 GPs. We excluded 88 who could not be assigned a practice (e.g., did not provide a valid practice code, worked in a private practice or in more than 1 practice) and 49 because the quota for their practice type had already been met. Of the remaining 330 interested GPs, 220 (67%) participated, but 4 (2%) did not complete. The final sample thus comprised 216 GPs, 54 from each practice type (LL, LH, HL, HH). Over half were female (58.33%, 126/216), and most were GP partners (67%, 145/216), the rest being salaried GPs. In total, 151 practices were represented in our sample, and the number of GPs working in the same practice ranged from 1 to 5 (median, 1).

Experience ranged from 2 months to 36 years in general practice postqualification (median 10 years, interquartile range >6 and <17.5 years) and was positively skewed (skewness 0.83). Due to the skewness, we categorized experience in 4 groups with cutoffs at 25%, 50%, and 75% of the distribution: <6 years (61 GPs, group 1), 6 to 10 years (52 GPs, group 2), 11 to 17.5 years (49 GPs, group 3), and 18 to 36 years (54 GPs, group 4). Table 1 presents performance measures for the whole sample and by experience group. GPs correctly referred 46.4% of the positive cases (2204/4752) and correctly rejected 77.2% of the negative cases (3668/4752; Table 2).

**Table 1:** Mean Number of Urgent Referrals, Hits, Misses, False Alarms (FAs), and Correct Rejections (CRs); Mean Discrimination (*d* ′); and Mean Criterion (*c*) of the Whole Sample of 216 GPs and by Experience Group

Performance Measure	Whole Sample, Mean (SD)	Experience Groups (Years in General Practice), Mean (SD)			
		Group 1 (0–6 Years)	Group 2 (7–10 Years)	Group 3 (11–17 Years)	Group 4 (18–36 Years)
Urgent referrals	15.22 (9.97)	16.23 (9.58)	16.36 (10.05)	15.06 (8.96)	13.13 (11.07)
Hits	10.20 (5.77)	10.98 (5.44)	10.86 (5.89)	10.45 (5.55)	8.46 (5.98)
Misses	11.80 (5.77)	11.01 (5.44)	11.13 (5.89)	11.55 (5.55)	13.54 (5.98)
FAs	5.02 (4.57)	5.25 (4.49)	5.50 (4.46)	4.61 (3.82)	4.67 (5.39)
CRs	16.98 (4.57)	16.75 (4.49)	16.5 (4.46)	17.39 (3.82)	17.33 (5.39)
*d* ′	.77 (.36)	.83 (.35)	.77 (.34)	.83 (.38)	.66 (.37)
*c*	.50 (.75)	.41 (.70)	.43 (.77)	.51 (.68)	.67 (.84)

**Table 2:** Frequencies of Referral and Nonreferral Decisions across the 9504 Responses (216 GPs Responding to 44 Vignettes)

	Positive Cases, No.	Negative Cases, No.	Total, No.
Urgent referrals	2204	1084	3288
No urgent referrals	2548	3668	6216
Total	4752	4752	9504

### Discrimination (*d* ′)

**Figure 3:**
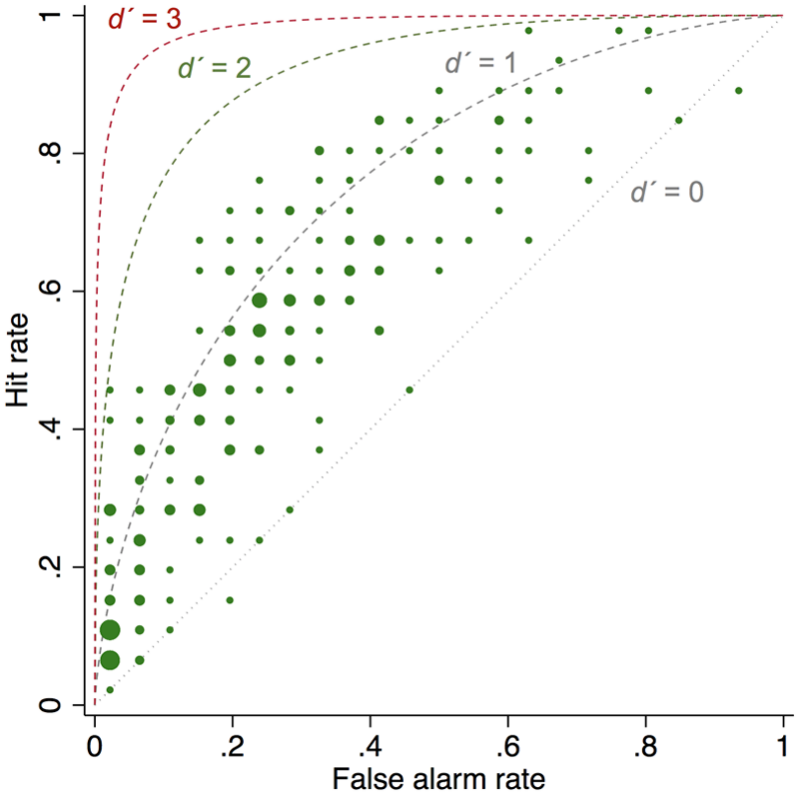
Scatterplot of the hit and false alarm rates of the 216 general practitioners (GPs), with superimposed theoretical receiver operating characteristic curves produced by *d* ′ = 0, *d* ′ = 1, *d* ′ = 2, and *d* ′ = 3. The dots of the scatterplot are sized by frequency (number of GPs with the same hit and false alarm rates).

**Figure 4:**
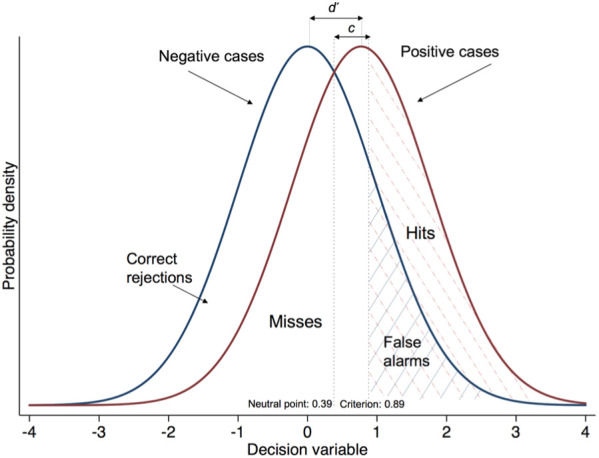
Distribution of the decision variable across positive and negative case presentations based on the standardized hit and false alarm rates of the 216 general practitioners. The distribution for negative cases has a mean of 0 and that for positive cases a mean of 0.77 (i.e., the separation is 0.77, which is equal to the sample’s *d* ′). The sample’s *c* is 0.50, which is the distance between the neutral point (0.77/2 = 0.39) and the criterion location (0.39 + 0.50 = 0.89).

The sample’s mean (SD) *d* ′ was 0.77 (0.36) and ranged from −0.28 to 1.91 (median, 0.78). Two respondents had negative values, and 8 had *d* ′ of 0. Larger values of *d* ′ indicate better discrimination. Zero values indicate chance performance expected from random guesses. Negative values may arise due to response confusion (the respondent means to say “yes” but selects “no” and vice versa).^18^ Both negative and zero values could suggest inattention and lack of engagement with the task. In Figure 3, the GPs with *d* ′ = 0 can be clearly seen lying along the diagonal line of the receiver operating characteristic (ROC) curve, where the hit and the false alarm rates are equal; the 2 GPs with negative *d* ′ can be seen below the diagonal. Figure 4 operationalizes SDT using the sample’s average performance and can be compared with Figure 1, where *d* ′ = 2.

The area under the ROC curve (AUC) is an alternative measure of discrimination. To calculate it, we conducted a probit regression with case type (positive v. negative) as predictor of the referral decision. The AUC was 0.63, suggesting that if our GPs were presented with a pair of cases, one positive and one negative, on a series of trials, they would refer the positive case on 63% of trials on average.

In univariate models, regressing *d* ′ on practice PPV, practice sensitivity, and GP gender revealed no association. Regressing *d* ′ on GP experience detected an association. Specifically, groups 1 and 3 showed significantly higher discrimination than the most experienced group (*b* = 0.17 [0.03–0.30], *P* = 0.015, for groups 1 v. 4 and *b* = 0.16 [0.02–0.30], *P* = 0.022, for groups 3 v. 4).

### Criterion (*c*)

The sample’s mean (SD) *c* was 0.50 (0.75) and ranged from −1.44 to 2.02 (median, 0.43). Positive values of *c* indicate bias toward “no” responses. Univariate regressions of *c* on practice characteristics and GP demographics detected significant associations with only 2 of the predictors of interest: practice PPV and GP gender. In the multivariable regression with these 2 predictors, practice PPV was positively associated with *c* (*b* = 0.06 [0.02–0.09], *P* = 0.001): as practice PPV increased, GPs’ *c*also increased, suggesting a more conservative approach to referral. Thus, only our first hypothesis (hypothesis 1) was confirmed. The model also detected a relationship with gender (*b* = −0.20 [–0.40 to −0.001], *P* = 0.049): female GPs had lower *c* than their male counterparts (mean *c*, 0.41 v. 0.62 for females v. males, respectively).^ii^

The associations of *c* with practice PPV and GP gender remained robust when the analyses were repeated with different risk thresholds (see online Appendix 3). These analyses also provided further support for an inverse relationship between discrimination and GP experience.

### Symptoms and Risk Factors That Predicted Misses and False Alarms

Table 3 shows the results from the multilevel regression models for misses and false alarms separately. Older patients and smokers with more pack years who presented with cough, weight loss, raised platelets, or appetite loss were more likely to be referred urgently. Smoking, cough, weight loss, and raised platelets significantly reduced the odds of a miss and increased the odds of a false alarm, suggesting a consistent effect (when one type of error increases, the other reduces). Fatigue and breathlessness significantly increased the odds of a miss, while patient age significantly reduced them, with no effect on false alarms. Appetite loss increased the odds of both types of error.

**Table 3:** Results (Odds Ratio [95% Confidence Interval], *P* Value) of the Multilevel Mixed-Effects Logistic Regression Models Predicting Misses and False Alarms Separately^a^

	Misses	False Alarms
Patient age	0.97 [0.96–0.99], *P* = 0.003	1.00 [0.98–1.02], *P* = 0.791
Smoking status		
Ex-smokers v. never-smokers	**0.35 [0.24–0.52], *P* < 0.001**	**2.21 [1.57–3.11], *P* < 0.001**
Current v. never-smokers	**0.31 [0.18–0.54], *P* < 0.001**	**2.21 [1.45–3.39], *P* < 0.001**
Pack years	1.00 [0.99–1.00], *P* = 0.512	1.02 [1.01–1.03], *P* = 0.001
**Cough**	**0.72 [0.58–0.89], *P* = 0.003**	**5.22 [3.45–7.88], *P* < 0.001**
**Weight loss**	**0.39 [0.29–0.54], *P* < 0.001**	**38.13 [22.60–64.34], *P* < 0.001**
**Raised platelets**	**0.36 [0.26–0.48], *P* < 0.001**	**11.47 [6.59–19.95], *P* < 0.001**
Fatigue	2.09 [1.63–2.69], *P* < 0.001	1.31 [0.87–1.96], *P* = 0.192
Breathlessness	2.79 [2.15–3.62], *P* < 0.001	1.18 [0.67–2.06], *P* = 0.571
Appetite loss	1.94 [1.49–2.52], *P* < 0.001	2.26 [1.44–3.55], *P* < 0.001

a Bold font indicates that the variable has a statistically significant effect *and* behaves consistently across models (increasing the likelihood of one type of error and decreasing the likelihood of the other). Coding notes: Model predicting misses: 1 = misses, 0 = hits. Model predicting false alarms: 1 = false alarms, 0 = correct rejections. Smoking status: 0 = never-smoker, 1 = ex-smoker, 2 = current smoker. Pack years: (number of cigarettes per day/20) * years of smoking. Pack years for never-smokers = 0. Symptoms: 0 = absent, 1 = present.

#### Five most frequent misses

Three of the positive cases most frequently missed (511 times) presented with breathlessness. The most common working diagnosis was a chronic respiratory disorder (75%, 382/511; e.g., chronic obstructive pulmonary disease), and the second most common was a heart condition (18%, 91/511; e.g., heart failure). Cancer was mentioned only 11% of the time as a differential (56/511). The other 2 positive cases most frequently missed (334 times) presented with appetite loss. Different working diagnoses were put forward, including gastric (14%, 48/334) and psychological (16%, 53/334) causes. Most frequently, however, GPs were too uncertain to give a diagnosis (35%, 118/334). Cancer was recorded as a differential 28% of the time (94/334).

#### Five most frequent false alarms

Three of the negative cases most frequently referred (336 times) presented with weight loss. Cancer was the working diagnosis in most cases (98%, 330/336). The other 2 false alarms presented with raised platelets; they were referred 182 times. Cancer was the working diagnosis in 99% of these cases (180/182).

## Discussion

This is the first study to associate practitioner decision making with organizational performance in cancer referral decisions. We found a strong relationship between practice referral PPV (chance of urgent referrals identifying lung cancer) and GPs’ decision making in cases of possible lung cancer, presented as brief clinical vignettes. Employing a signal detection approach allowed us to disentangle this relationship and attribute it to response bias—the GPs’ inclination to refer—rather than their ability to discriminate those who should be referred from those who should not. A significant relationship between characteristics of the organization and physicians’ response bias has been reported before: a study of 168 US emergency physicians responding to 30 clinical vignettes of trauma cases found that physicians at hospitals with a trauma center affiliation were more inclined to transfer trauma patients than those at hospitals without an affiliation.^8^

The sample’s average discrimination was above chance but modest (*d* ′ = 0.77) and highly variable, ranging from −0.28 to 1.91. Values of *d* ′ reported in the literature vary between domains, with a *d* ′ of 4 considered impressive and unattainable by unaided human judgment.^21^ We found the lowest average discrimination in the most experienced group (GPs with 18 or more years in practice). This significant difference from the less experienced groups was robust to different risk thresholds (online Appendix 3). However, we cannot draw any strong conclusions from the data, as we did not seek to recruit a representative sample of GPs of different experience. The range of years in practice was much larger in the most experienced group than in the other 3 groups. More evidence is required.

Due to the dearth of published evidence, we could not develop richer patient descriptions and quantify their cancer risk; our vignettes contained 2 symptoms at most and no qualifying information that a GP would normally elicit (e.g., how often, how bad, what makes it better or worse). We based our vignettes on a population case-control study that used both coded and free-text data from primary care patient records—therefore, the richest information available. Providing additional information to GPs could improve but also reduce discrimination (e.g., by focusing attention to irrelevant or non-evidence-based information). Having more vignettes with a clearer signal (e.g., hemoptysis or abnormal chest X-ray) would certainly improve discrimination but would not reflect the real-life decision difficulty that GPs experience.

We found no relationship between practice referral sensitivity (chance of cancer patients being picked up via urgent referral from their practice) and GP performance in the study. It is possible that sensitivity is influenced more by patient rather than GP behavior. For example, Meechan and colleagues^2^ reported lower sensitivity in the most deprived areas. There is evidence that patients in these areas are more likely to be diagnosed as emergency presentations rather than following presentation to the GP,^22^ thereby directly reducing the practice’s referral sensitivity by increasing the miss rate (but not affecting the practice PPV).

Our results suggest that GPs are aware of some of the symptoms included in the latest NICE guidelines as indicative of lung cancer. Smoking, cough, weight loss, and raised platelets made urgent referrals more likely, reducing the likelihood of misses and increasing the likelihood of false alarms. In contrast, breathlessness increased misses by suggesting other, noncancer diagnoses. Appetite loss increased both types of error. We hypothesize that the effect of appetite loss depends on the context: when it presents either on its own or in association with another nonspecific symptom (e.g., fatigue), it increases uncertainty and can lead to misses; when it presents with a red flag (e.g., raised platelets), it is the red flag that drives the decision.

Female GPs exhibited a more lenient approach to referral on average compared to males. This difference has been reported before in emergency medicine.^8^ A lenient approach to referral may reflect a wish to reduce anxiety due to uncertainty. Differences have been found between male and female physicians, with females reporting greater anxiety due to uncertainty.^23^ A lenient approach may also reflect greater risk aversion. Sex-related differences have been found in risk attitudes, with women reporting willingness to take fewer risks than men and seeing negative outcomes as more likely and more severe.^24–26^

In summary, our study highlights that a high referral PPV at the practice level indicates a conservative approach to referral by individual GPs, and it is unrelated to the GPs’ discrimination ability. At the same time, referral sensitivity at the practice level appears unrelated to GP decision making. Therefore, a combination of high referral PPV with high sensitivity does not necessarily indicate “good clinical practice,” as it has been claimed.^2^ Our study illustrates why it is important to decompose performance to its 2 constituent parts, discrimination and response bias.

We would argue that a more lenient approach to referral, *only* when combined with better evidence to support referral decisions, could improve lung cancer outcomes. Blunt incentives or mandates to either increase or reduce referral rates without better evidence will inevitably inflate one type of error—either misses or false alarms. The question is whether we can estimate more accurately a patient’s lung cancer risk by combining prior risk data (age, smoking history, comorbidity) and current symptoms and, importantly, whether GPs can use these risk estimates for improved decision making at the point of care. Given the low to moderate diagnostic value of the current evidence, producing accurate risk estimates would necessitate a concerted effort to capture more and better symptom data in routine consultations, where currently only 1.6 data items are coded on average.^27^ We recently demonstrated the feasibility of combining diagnostic decision support with improved coding of symptoms and signs in the health record.^27^ This study adds weight to the argument for developing a learning system for cancer diagnosis (involving continuous cycles of collecting and mining routine, coded data to generate new prediction rules for decision support)^28^ as a means of addressing performance variation in the referral of patients with suspected cancer.

## Supplemental Material

DS_10.1177_0272989X18813357 – Supplemental material for Referral Decision Making of General Practitioners: A Signal Detection Study Supplemental material, DS_10.1177_0272989X18813357 for Referral Decision Making of General Practitioners: A Signal Detection Study by Olga Kostopoulou, Martine Nurek, Simona Cantarella, Grace Okoli, Francesca Fiorentino and Brendan C. Delaney in Medical Decision Making

## Notes

i. Another measure of response bias is beta (β), which assumes that responses are based on a likelihood ratio: the likelihood of obtaining a value x on the decision variable during a signal trial (the height of the signal distribution at *x*) over the likelihood of obtaining that value during a noise trial (the height of the noise distribution at *x*). Beta is a ratio; therefore, its natural logarithm is used for statistical analyses.^18^

ii. (Repeating the regression analyses on the other measure of response bias, ln(β), returned a significant relationship only with practice PPV, *b* = 0.03 (0.002-0.06), *P* = 0.039.
