# Supplementary material for: Referral Decision Making of General Practitioners: A Signal Detection Study
Source: Med Decis Making. 2018 Dec 27;39(1):21–31. doi: 10.1177/0272989X18813357 (PMC6311616; doi:10.1177/0272989X18813357)
Supplement: DS_10.1177_0272989X18813357 – Supplemental material for Referral Decision Making of General Practitioners: A Signal Detection Study [file DS_10.1177_0272989X18813357.pdf]

Appendix 1: Adjusting the NICE risk threshold (0.03) to account for a negative chest X-ray result

The data come from Sobue et al. (1991), table IV, p. 1072.

		Cancer		
		**+**	**-**	Total
**Chest X-ray**	**+**	42	1307	1349
	**-**	32	32109	32141
	Total	74	33416	33490

On the basis of the table, we calculate the following values:

Sensitivity = 0.56756757

Specificity = 0.960887

LR+ = sens/1-spec = 14.51097

LR- = 1-sens/spec = 0.45003464

We converted the NICE risk threshold to odds (0.0309). We then used the equation Post-test odds=prior odds * LR, and solved it for prior odds (prior odds = 0.0309 / 0.45). This gives prior odds of 0.0687, which we converted to probability (0.064). Thus, a new adjusted risk threshold of 6.43% was estimated, to account for a normal chest X-ray.

Reference: Sobue T, Suzuki T, Matsuda M, et al. Sensitivity and Specificity of Lung Cancer Screening in Osaka, Japan. *Japanese J Cancer Res*. 1991;82(10):1069-1076. doi:10.1111/j.1349-7006.1991.tb01759.x

Appendix 2: A GLM-based estimation of SDT

GLMs can be used to estimate SDT parameters and to test the influence of covariates on these parameters. GLMs map the probability *p* of a categorical response to a linear predictor η with a link function. For SDT, it is common to use either a logit or a probit (inverse normal) link function (DeCarlo, 1998; Wright et al. 2009). In a probit GLM, $p = \Phi(\eta)$, where Φ is the cumulative distribution function of the standard normal distribution that maps probabilities to z scores. The linear predictor η is modelled on an intercept and a slope: $\eta = \beta_0 + \beta_1 X$. The intercept is the standardised probability of saying 'yes' when noise is presented (*z*FA), and equals -*k*, where $k = c + d'/2$, i.e., the distance from the mean of the noise distribution (MacMillan and Creelman, 2005, p. 339). The slope is the increase in the z-score of saying 'yes' when signal is presented, and equals *d*´ (https://vuorre.netlify.com/post/2017/bayesian-estimation-of-signal-detection-theory-models-part-1/).

We conducted a mixed effects probit regression with random intercept for GPs and random slopes for case type, thus allowing GPs to differ both in their response bias (random intercept) and discrimination (random slopes). The table below presents the model estimates for the slope (0.83) and the intercept (-0.95), which give *z*(H) = -0.12 and *z*(FA) = -0.95. Converting into probabilities, we get *p*(H) = 0.45, and *p*(FA) = 0.17, which match the mean hit and false alarm rates of the data (0.45 and 0.23 respectively). From the intercept -*k*, we calculate criterion *c* as = 0.95 - (0.83/2) = 0.53, which matches the estimate reported in the paper.

	Coefficient	95% CI	P
Case type (negative=0, positive=1)	0.83	0.77 to 0.89	0.000
Constant	-0.95	-1.07 to -0.84	0.000

Table 1. Probit regression coefficients (95% CI and P values) for predicting response probability (refer vs. not refer) as a function of case type (positive vs. negative)

We entered predictors in the model (practice PPV – mean-centered – and GP gender) and their interactions, to see their simultaneous effect on discrimination and criterion, and to compare with the results from the traditional SDT calculations presented in the paper. The estimates are presented in the table below. The main effects are significant and similar to those reported in the paper: increasing practice PPV reduces the probability of referral (b = -0.07 [-0.11 to -0.03] *P*=0.001). Female GPs are more likely to refer than male GPs (b = 0.25 [0.02 to 0.47] *P*=0.033).

In sum, the conclusions drawn from the GLM-based and the traditional SDT estimation methods are comparable.

	Coefficient	95% CI	P
Case type (negative=0, positive=1)	0.85	0.75 to 0.95	0.000
C_PPV	-0.07	-0.11 to -0.03	0.001
Case type * C_PPV	0.009	-0.01 to 0.03	0.403
Gender (male=0, female=1)	0.25	0.02 to 0.47	0.033
Case type * gender	-0.04	-0.16 to 0.09	0.578
Constant	-1.10	-1.28 to -0.93	0.000

Table 2. Probit regression coefficients (95% CI and P values) for predicting response probability (refer vs. not refer) as a function of case type (positive vs. negative), practice PPV (mean centered), GP gender, and interactions.

References

- DeCarlo LT. Signal detection theory and generalized linear models. *Psychol Methods*. 1998;3(2):186-205. doi:10.1037//1082-989X.3.2.186.
- Macmillan N, Creelman C. *Detection Theory: A User's Guide*. 2nd ed. New York: Lawrence Erlbaum Associates Inc.; 2005.
- Wright, D. B., Horry, R., & Skagerberg, E. M. (2009). Functions for traditional and multilevel approaches to signal detection theory. Behavior Research Methods, 41(2), 257–267. http://doi.org/10.3758/BRM.41.2.257

Appendix 3: Regression analyses using different risk thresholds

Each time that we used a different risk threshold, the number of positive and negative cases changed, which resulted in different hit and false alarm rates, and different *d´* and *c*. The table below presents the results. In summary, our two main findings (*c* being predicted by practice PPV, and *c* being significantly higher in male than female GPs) remained unchanged. Depending on the threshold used, we detected two inverse relationships between *d´* and practice PPV, and one between *d´* and practice sensitivity. Regressing *d´* on GP experience returned a significant negative relationship (*d´* declining with experience) for two of the thresholds (the 3% and the threshold using the upper 95% confidence interval of the likelihood ratios – see table); and significant differences between the most experienced group and groups 1 and 3 for the other two thresholds (6.43% and the threshold using the lower 95% confidence interval of the likelihood ratios – see table), all showing lower *d´* in the most experienced group. Except for one case of a significant difference between groups 1 and 4 when using the threshold based on the lower 95% confidence interval, regressing *c* on GP experience did not return any other significant relationships (regression results are not presented in the table).

	Original threshold of 6.43%	NICE threshold of 3%	Threshold estimated with lower CI of LRs	Threshold estimated with upper CI of LRs
Number of negative and positive cases	22 Neg and 22 Pos	10 Neg and 34 Pos	37 Neg and 7 Pos	13 Neg and 31 Pos
Hit rate	0.46 (0.25)	0.40 (0.24)	0.38 (0.26)	0.42 (0.25)
False alarm rate	0.24 (0.20)	0.20 (0.16)	0.35 (0.21)	0.20 (0.17)
d´	0.77 (0.36)	0.68 (0.52)	0.10 (0.43)	0.77 (0.42)
c	0.50 (0.75)	0.66 (0.67)	0.44 (0.74)	0.64 (0.70)
Regression of *c* on practice PPV	b=0.06 [0.02-0.09] *P*=0.001	b=0.05 [0.02-0.08] *P*=0.001	b=0.06 [0.03-0.10] *P*<0.001	b=0.06 [0.02-0.09] *P*=0.001
Regression of *c* on practice sensitivity	NS	NS	NS	NS
Regression of *d´* on practice PPV	NS	b= -0.03 [-0.05 to -0.003] *P*=0.03	b= -0.02 [-0.04 to -0.0007] *P*=0.04	NS
Regression of *d´* on practice sensitivity	NS	NS	b= -0.01 [-0.02 to -0.003] *P*=0.006	NS
Regression of *c* on GP gender	b= -0.21 [-0.42 to -0.011], P=0.039	b= -0.02 [-0.038 to -0.022], P=0.028	b= -0.21 [-0.41 to -0.014], P=0.036	b= -0.20 [-0.38 to -0.010], P=0.042
Regression of *d´* on GP experience	1. b=0.17 [0.03-0.30] *P*=0.02<br>2. b=0.10 [-0.04-0.24] *P*=0.15<br>3. b=0.16, [0.02-0.30] *P*=0.02	1. b=0.40 [0.22-0.59] *P*<0.0001<br>2. b=0.21 [-0.02-0.40] *P*=0.03<br>3. b=0.15, [-0.05 to 0.34] *P*=0.14	1. b=0.15 [-0.005-0.31] *P*=0.06<br>2. b=0.03 [-0.14-0.19] *P*=0.73<br>3. b=0.16, [-0.007-0.33] *P*=0.06	1. b=0.35 [0.20-0.50] *P*<0.0001<br>2. b=0.19 [0.04-0.35] *P*=0.01<br>3. b=0.18, [0.02-0.33] *P*=0.03

Table. Results of univariate regression analyses using different risk thresholds. The last row presents regressions of *d´* on GP experience, with group 4 (18-36 years in practice) as the reference category; the three regression coefficients refer to the comparisons between group 4 and groups 1, 2, and 3 respectively.
